# Bacterial community and chemical profiles of oil-polluted sites in selected cities of Uganda: potential for developing a bacterial-based product for remediation of oil-polluted sites

**DOI:** 10.1186/s12866-022-02541-x

**Published:** 2022-05-03

**Authors:** Jamilu E. Ssenku, Abdul Walusansa, Hannington Oryem-Origa, Paul Ssemanda, Saidi Ntambi, Francis Omujal, Abubakar Sadik Mustafa

**Affiliations:** 1 Department of Plant Sciences, Microbiology & Biotechnology, Makerere University, Kampala, Uganda; 2 Department of Microbiology, Faculty of Health Sciences, Islamic University in Uganda, Kampala, Uganda; 3 Department of Medical Microbiology and Immunology, Faculty of Health Sciences, Busitema University, Mbale, Uganda; 4 Department of Biology and Chemistry, Universität Bremen, Bremen, Germany; 5 Department of Chemistry, Natural Chemotherapeutics Research Institute (NCRI), Kampala, Uganda

**Keywords:** Bacterial profiles, BIOLOG EcoPlate™, Bioremediation, Chemical profiles, Garages, Hydrocarbons, Industries, Oil-pollution

## Abstract

**Background:**

Oil spills are ranked among the greatest global challenges to humanity. In Uganda, owing to the forthcoming full-scale production of multi-billion barrels of oil, the country’s oil pollution burden is anticipated to escalate, necessitating remediation. Due to the unsuitability of several oil clean-up technologies, the search for cost-effective and environmentally friendly remediation technologies is paramount. We thus carried out this study to examine the occurrence of metabolically active indigenous bacterial species and chemical characteristics of soils with a long history of oil pollution in Uganda that can be used in the development of a bacterial-based product for remediation of oil-polluted sites.

**Results:**

Total hydrocarbon analysis of the soil samples revealed that the three most abundant hydrocarbons were pyrene, anthracene and phenanthrene that were significantly higher in oil-polluted sites than in the control sites. Using the BIOLOG EcoPlate™, the study revealed that bacterial species richness, bacterial diversity and bacterial activity (ANOVA, *p* < 0.05) significantly varied among the sites. Only bacterial activity showed significant variation across the three cities (ANOVA, *p* < 0.05). Additionally, the study revealed significant moderate positive correlation between the bacterial community profiles with Zn and organic contents while correlations between the bacterial community profiles and the hydrocarbons were largely moderate and positively correlated.

**Conclusions:**

This study revealed largely similar bacterial community profiles between the oil-polluted and control sites suggestive of the occurrence of metabolically active bacterial populations in both sites. The oil-polluted sites had higher petroleum hydrocarbon, heavy metal, nitrogen and phosphorus contents. Even though we observed similar bacterial community profiles between the oil polluted and control sites, the actual bacterial community composition may be different, owing to a higher exposure to petroleum hydrocarbons. However, the existence of oil degrading bacteria in unpolluted soils should not be overlooked. Thus, there is a need to ascertain the actual indigenous bacterial populations with potential to degrade hydrocarbons from both oil-polluted and unpolluted sites in Uganda to inform the design and development of a bacterial-based oil remediation product that could be used to manage the imminent pollution from oil exploration and increased utilization of petroleum products in Uganda.

**Supplementary Information:**

The online version contains supplementary material available at 10.1186/s12866-022-02541-x.

## Introduction

With the enormous use of petroleum hydrocarbons in commercial manufacturing and transportation, mineral oil has turn out to be a vital fossil gas worldwide [1]. Consequently, one of the world’s commonest environmental challenge is caused by pollution of toxic petroleum products including heavy metals, benzene series and polycyclic aromatic hydrocarbons (PAHs) [1–5]. Due to the diverse chemical composition of petroleum products, its continuous contamination of the environment causes both short and long-term side effects [5]. Toxic petroleum products have been associated with numerous threats to human life and environment which includes alteration of soil characteristics, ecological dysfunction, reduced agricultural productivity and increased risk to human health [6–10].

Oil pollution of soil affects ecological processes leading to changes in microbial communities and activities within the soil [11, 12]. The adaptations and genetic changes shift towards hydrocarbon-degrading microorganisms [13]. Isolation of high numbers of some microorganisms, especially fungi and bacteria, from oil polluted sites serves to affirm their potential to utilize the pollutant [14–16]. These isolates can play an important role in remediation of oil contaminated sites through bioremediation techniques [17].

The adverse impacts of oil contamination on surrounding environments as well as health [9, 18] calls for urgency of survey, extraction and removal of oil contaminants from soil [19]. Several clean-up technologies have been reported on the removal of hydrocarbons in polluted soils but most of them are either very expensive or require the integration of advanced mechanization [20]. Biodegradation by natural populations of microorganisms represents one of the primary mechanisms by which petroleum and other hydrocarbon pollutants can be eliminated from the environment [21]. The technique involves the stimulation of specific microbes that use the discharged petroleum contaminants as a source of food and energy. Contrary to the several clean-up technologies for petroleum hydrocarbons in contaminated soils, the bioremediation technology has been reported to be efficient, cost-effective and environmentally friendly [20].

In earlier bioremediation studies by Li,Sun [22], indigenous microbes were found to be very effective in the degradation of total petroleum hydrocarbons than the introduced exotic microbes. Furthermore, bioremediation through bioaugmentation has always been unsuccessful [23], owing to the failure of the introduced exogenous microbes to compete favorably with the indigenous microbes at the polluted site [24, 25] probably due to the site condition and ecological specificity of the polluted area [23]. Consequently, bacteria were recently reported as the most active agents in oil bioremediation [26–28]. Therefore, there are prospects for the development of bacterial-based oil-remediation products from the microbes present in the oil-polluted soil samples from the current study.

With the anticipated full-scale production of Uganda’s 6.0 billion barrels of proven oil reserves in the Albertine region [29] and the unregulated dumping of used oil products into the environment, oil and petroleum product pollution is likely to escalate. Thus, searching for more effective and more environmentally friendly bioremediation techniques involving the use of indigenous microbes is paramount. To the best of our knowledge, no baseline studies aimed at confirming the existence of petroleum hydrocarbon-degrading microbes in polluted soils in Uganda has been done. This study was carried out in selected garages and industries with a long history of motor oil pollution across the country to ascertain the occurrence of metabolically active bacterial populations. Establishing the occurrence of metabolically active bacterial community in oil-polluted sites would pave way for isolation and characterization of indigenous bacterial populations to be assessed for oil-biodegradability potential and development of a bacterial-based product for remediation of oil polluted sites.

## Materials and methods

### Study site description

**Fig. 1:**
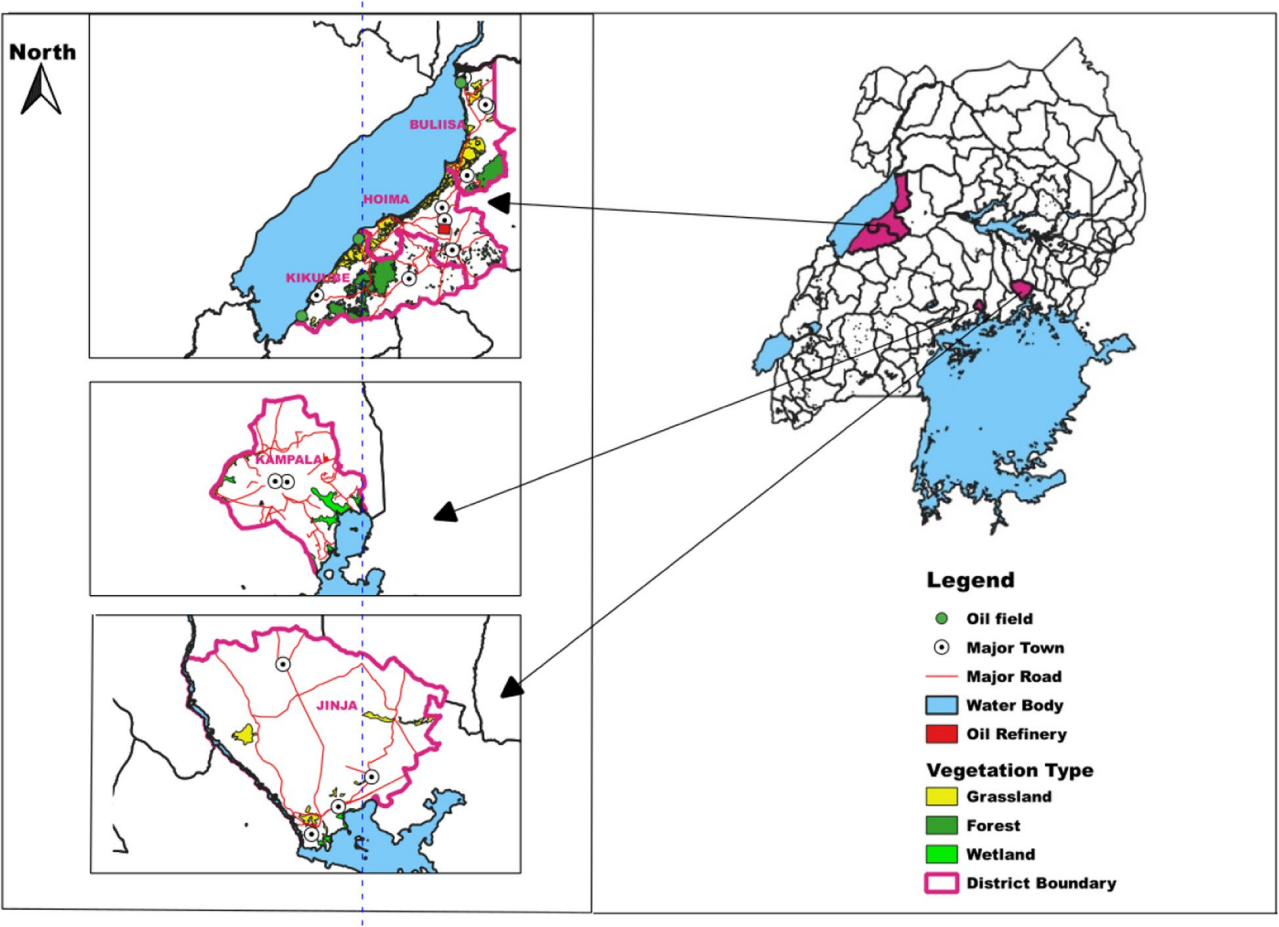
Location of the study sites

The study was carried out in three cities, namely Kampala, Jinja and Hoima, located in the Central, Eastern and Western regions of Uganda, respectively (Fig. 1). The three cities lie within the geographical coordinates of 0° 18′ 58.61“ N and 32° 34’ 55.88” E (Kampala), 0° 26′ 20.47“ N and 33° 12’ 11.41” E (Jinja) and 1° 25′ 59.30“ N and 31° 21’ 8.68” E (Hoima) and at an average elevation of 1211, 1204 and 1071 m above sea level, respectively. They experience a tropical climate receiving an annual rainfall of 999.9, 2015 and 1435 mm for Kampala, Jinja and Hoima, respectively, and rainfall is bi-modally distributed with the wetter periods occurring from March to May and August to November. They experience a mean minimum temperature of 18.5 °C and a mean maximum temperature of 30.8 °C.

### Sampling technique

Soil samples were collected from purposively selected sites within garages and industries with a long history of oil pollution. For each of the oil-polluted garage or industry, five spots were strategically selected and samples were collected within a depth of 0 to 15 cm using a stainless steel soil auger and trowel. Upon completion of sampling from a particular site, the trowel and the soil augur were sterilized by dipping them in 70% ethanol and burning to avoid cross contamination of samples. For each site, the five samples were mixed thoroughly within a polythene bag to constitute a composite sample. Similarly, control soil samples were picked from the nearby unpolluted sites, 1000 m away from the polluted sites [30]. For each site, 500 g of the composite sample was transferred into well-labeled sterile zip-lock bag, immediately sealed off and transferred into a cold box maintained at 4 °C to avoid any changes in the bacterial community structure and the chemical characteristics of the samples. The samples were transported to the Central Diagnostics Laboratory at the Department of Plant Sciences, Microbiology and Biotechnology, Makerere University.

### Determination of the chemical characteristics of the soil samples

The soil samples were oven dried at 40 °C for 24 hrs [31]. The dry soil samples were later ground to a fine powder using a ceramic mortar and pestle and sieved with a 500 μm pore size metallic sieve (Endecotts BS410, Fisher Scientific UK) to remove coarse particles and debris. Soil pH was determined using a calibrated pH meter, organic matter content by Walkley-Black potassium dichromate wet oxidation method [32] while total nitrogen was determined by the semi-micro Kjeldahl method [33]. The available phosphorus was determined following Ammonium Molybdate-Ascorbic acid method [34], using a UV/Visible spectrophotometer at 860 nm.

### Determination of heavy metal content of the soil samples

Soils were analyzed for total concentration of Zn, Cr, Mn, Ni and Pb at the Chemistry Analytical Laboratory, Department of Chemistry, Makerere University. A fine ground dry soil sample of 1.25 g was weighed and transferred to a 250 mL conical flask and 50 mL of deionized water was added, followed by 50 mL of *aqua regia*, a mixture of concentrated HCl and HNO_3_ AR 70% (Fisher Scientific, UK), in a ratio of 3:1. The resultant mixture in the conical flask was placed on a hot plate inside a fume cupboard, heated at a temperature of 100 °C for 1 hour, and then at 125 °C, 150 °C, and 175 °C at intervals of 15 min until 5 mL of the mixture was left in the conical flask. This was followed by cooling to room temperature before adding 1 mL of 30% H_2_O_2_ and heating for another 15 min. This procedure was repeated once, and then 3 mL of 30% H_2_O_2_ was added and the mixture heated for 15 min to complete the digestion of any organic matter. The digested sample was cooled and diluted with deionized water up to the 100 mL mark before filtering for analysis of total concentration of the heavy metals with Flame Atomic Absorption Spectrophotometer (Agilent 240FS AA, USA).

### Determination of the total hydrocarbons

#### Sample extraction

Determination of total hydrocarbon contents of the samples was done at the Directorate of Government Analytical Laboratory (DGAL), Ministry of Internal Affairs, Kampala, Uganda. Extraction of samples was done according to Coulon and Wu [35] and Adeniji,Okoh [36]. Briefly, the soil samples in the amber bottle were thoroughly mixed to make a homogenous sample. Approximately 5 g of the soil composite was quickly weighed into a 100 mL-capacity amber bottle. Five grams of anhydrous NaSO_4_ was then added to the bottle and the mixture was agitated. An internal standard, 1-chlorooctadecane (300 μg/mL) was added to the soil sample and 30 mL of dichloromethane was added to the sample due to its consistency, efficiency and ability of not interfering with the samples. The bottle containing soil sample was corked very tight and transferred to a mechanical shaker. The sample was agitated for 6 hours at room temperature. After agitation, the sample was allowed to settle for 1 hour and then filtered through 110 mm filter paper in a Whatman fat-free extraction thimble into an Erlenmeyer flask. The filtrate was allowed to concentrate to 1 mL by evaporation overnight in a fume hood.

#### Sample cleaning

The extracted samples were cleaned using a glass column to remove all impurities in preparation for Gas Chromatography (GC) column analysis according to Alinnor and Nwachukwu [37] and Okop and Ekpo [38]. Briefly, the concentrated sample extract was mixed with cyclohexane in a beaker and transferred into prepared column. The column was packed with silica gel matrix using dichloromethane to form a slurry. Anhydrous Na_2_SO_4_ was added into the column followed by addition of pentane as an elution solvent. The sample extract put in the packed column was eluted using pentane as mobile phase solvent and the eluted sample was collected in a beaker. The sample was eluted further by adding more pentane through the column. After elution, the column was rinsed with dichloromethane. The eluted samples were left to stand overnight at room temperature in a fume hood for evaporation in order to concentrate the samples. A blank sample was simultaneously processed without the soil samples.

#### Gas chromatography-mass spectrophotometry analysis (GC-MS)

The separation and detection of compounds in the soil sample extracts and standards were carried out using Shimatzu TQ8040 triple Quadruple Gas Chromatography Tandem Mass Spectrophotometer according to Coulon and Wu [35], Okop and Ekpo [38] and Alinnor and Nwachukwu [37]. Briefly, 3 μl of concentrated cleaned eluted samples were put in vials and injected into GC-MS columns (−ZB-5SMi, 30 m × 0.25 mm × 0.25 μm 50 m × 0.32 mm) for separation of compounds. The micro-syringe was rinsed with the dichloromethane after each sample was injected. Temperature profile of the GC-MS were programmed as; column initial temperature of 80 °C, held for 20 min, followed by a temperature increase of 5 °C /min to 180 °C, held for another 5 min to 250 °C, and more 15 min to 310 °C. Injection temperature was 320 °C and injection mode was splitless, Carrier gas was helium with pressure of 79 kPa, total flow 50 mL/min, column flow − 1.29 mL/min and linear velocity- 41.4 mL/min. After separation, the compounds were passed through the mass spectrophotometer with electron impact (EI) ionisation carried out at 70 eV and the ion source temperature at 250 °C. Data from the GC–MS and compounds were identified based on comparison of mass spectral fragmentation with those in the NIST library. The total hydrocarbon content of the soil samples were resolved at a particular chromatogram in mg of total hydrocarbon/kg of soil sample.

#### Determination of bacterial activity

BIOLOG EcoPlate™ (Biolog, Inc., Hayward, CA, USA), which contains three replicated wells of 31 carbon substrates, was used to investigate the carbon metabolic activity among the aerobic and heterotrophic bacterial communities in all the soil samples [39–42]. Briefly, a soil suspension was prepared by vortexing 1.5 g of soil (dry weight) in 15 mL of sterile phosphate buffered saline (pH, 7) and allowed to settle for 2 hours. The supernatant was serially diluted up to 10^− 2^ dilution. Using a multi-channel pipette, aliquots of 150 μL of the 10^− 2^ dilution for each sample was inoculated into each well of BIOLOG Ecoplates™ and incubated at 28 °C in an oven. Colour development of each well was measured as the optical density immediately after inoculation and after every 24 hours post-inoculation for 1 week at 590 nm with the BioTeK microplate reader (ELx800TM, USA).

#### Analysis of bacterial activity

Individual absorbance values of the 31 single substrates were corrected by subtraction of the blank control value (raw difference). According to Classen,Boyle [43], the wells’ optical density values that were negative or under 0.06 to zero were adjusted. To minimize the effects of different inoculum densities, data were normalized by dividing the raw difference values by their respective average well colour development (AWCD) values. The number of active wells were determined as described by Li [44] quantifying the number of positive wells (> 0.06 absorbance units above the time zero reading) and this was taken to be a representative of the bacterial species richness [45]. The bacterial activity in each microplate was expressed as the average well colour development (AWCD) [46, 47] using the expression below, where *OD*_*1*_ is the optical density value from each well.

1 $$AWCD\kern0.5em =\kern0.5em \sum \frac{OD_i}{31}$$

The Shannon-Wiener Diversity Index (*H*) was used to determine the species diversity of bacteria as described by Yan,McBratney [48], Frąc,Oszust [46] and Fowler,Cohen [49] using the OD of 0.06 as threshold for positive response using the following expression:

2 $$H\kern0.5em =\kern0.5em \sum \limits_{i=l}^N pi\ln \kern0.5em pi$$

Where p_*i*_*,* in this case, is the proportion of AWCD of a particular substrate to the AWCD of all substrates of a particular soil sample.

#### Quality assurance

Prior to use, all solutions, transfer equipment, and glassware were sterilized in an autoclave. Weighing of soil samples, serial dilutions and plate inoculation was done under a laminar-flow hood to minimize the risk of contamination from the surrounding. For heavy metal analysis, the glassware used was thoroughly cleaned and all the reagents were of analytical grade. Double distilled water was used throughout the analysis. Replicate samples, blanks and standard reference were included in all analyses.

#### Data analysis

All statistical analyses were performed using the R statistical package 3.5.1 [50]. Data for community structure and activity were checked for normality of distribution and homogeneity of variance using Shapiro-Wilk test and then subjected to analysis of variance to explore variability across the selected cities and sites followed by separation of means by Tukey’s Honest Significant Multiple Comparison. Means were considered to be significantly different at *p* < 0.05. Data for species richness was transformed and analyzed using ANOVA but the median values were used to show the results. Median values and study ranges (lowest and highest values) were used to present the results for the chemical properties for each category of soil sample. The principle component analysis was used to explore the overall differences and similarities in bacterial community structure of the cities and sites.

## Results

### Chemical characterization of the oil polluted soils

The chemical characteristics of the soil samples collected are presented in Table 1 below (Supplementary Table 1). There was a wider variation (study range i.e. lowest-highest) in pH of the oil-polluted soil samples (5.3-7.9) as compared to the control soil samples (6.1-7.1). The organic matter content of the oil-polluted soil samples was 4.5 to 5.8 folds higher than that of the control soils. Generally, garage oil-polluted soil samples were richer in organic matter content than the industrial oil-polluted soil samples. We observed higher nitrogen and phosphorus in oil-polluted soil samples as compared to the control soil samples apart from soil samples collected in Kampala. The garage oil-polluted soils had higher heavy metal content than the control soil samples except for manganese collected from Jinja and nickel in soil samples collected from Kampala and Jinja. On the contrary, industrial oil-polluted soil samples had higher heavy metal content than the control soil samples except for chromium in soil samples collected from Jinja, manganese in soil samples collected from Kampala and Jinja, nickel in soil samples collected from Kampala and Jinja and lead in soil samples collected from Jinja.

**Table 1:** Chemical characteristics (median and range values) of soils samples collected from the three cities

Parameter	Site	Kampala city		Jinja city		Hoima city		Study ranges	
		Oil-polluted soil	Control	Oil-polluted soil	Control	Oil-polluted soil	Control	Oil-polluted soil	Control
pH	Garage	6.9	6.3	6.7	6.7	6.5	6.8	6.1-7.9	6.1-7.1
	Industry	7.9	6.3	7.0	6.7	6.7	6.8	5.3-7.3	6.1-7.1
Organic content (%)	Garage	25.5	4.50	26.9	4.20	20.3	3.50	14.0-43.8	1.6-6.0
	Industry	23.1	4.50	19.3	4.20	20.9	3.50	5.7-36.0	1.6-6.0
Nitrogen (%)	Garage	0.34	0.28	0.27	0.19	0.49	0.19	0.13 − 0.70	0.03 − 0.35
	Industry	0.22	0.28	0.32	0.19	0.34	0.19	0.08 − 0.60	0.03 − 0.35
Phosphorus (%)	Garage	0.27	0.24	0.17	0.07	0.08	0.06	0.06-0.88	0.01-0.33
	Industry	0.13	0.24	0.28	0.07	0.12	0.06	0.05-0.98	0.01-0.33
Zinc (mg/kg)	Garage	46.7	17.1	46.2	31.7	35.9	3.2	27.4-49.7	2.5-42.2
	Industry	24.8	17.1	36.9	31.7	37.4	3.2	6.4-48.6	2.5-42.2
Chromium (mg/kg)	Garage	19.1	9.6	17.8	17.3	16.5	8.5	7.5-30.4	4.6-25.5
	Industry	12.8	9.6	13.0	17.3	15.3	8.5	9.0-29.6	4.6-25.5
Manganese (mg/kg)	Garage	18.3	14.6	22.1	30.6	11.6	6.1	7.5-32.6	0.2-58.5
	Industry	9.8	14.6	25.5	30.6	13.7	6.1	5.0-50.3	0.2-58.5
Nickel (mg/kg)	Garage	0.42	0.43	0.75	1.57	0.37	0.14	0.12-2.25	0.03-3.3
	Industry	0.26	0.43	0.57	1.57	0.31	0.14	0.13-2.01	0.03-3.3
Lead (mg/kg)	Garage	1.51	0.31	2.01	1.99	0.19	0.07	0.06-3.99	0.04-4.01
	Industry	0.39	0.31	0.27	1.99	0.26	0.07	0.05-3.38	0.04-4.01

### Hydrocarbon contents of soil samples collected from the three cities

**Fig. 2:**
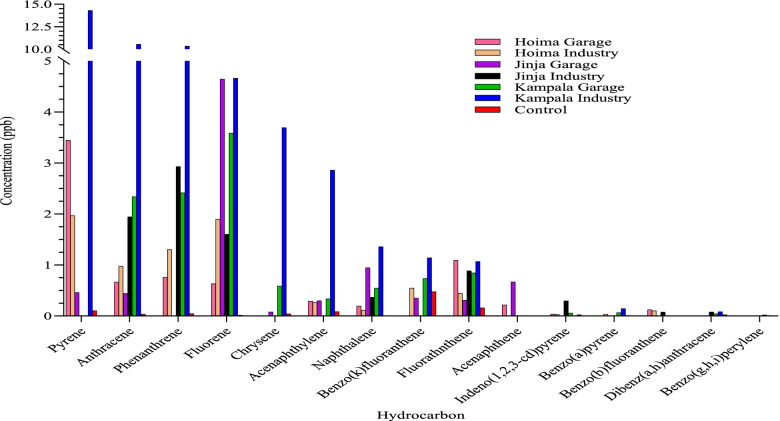
Most abundant hydrocarbons in soil samples collected from the three cities and sites

The hydrocarbon contents of the soil samples varied significantly between the cities (F_2,156_ = 117.88; *p* < 0.001) and the sites (F_1,156_ = 43.903; *p* < 0.001). The most abundant hydrocarbons were pyrene, anthracene, phenanthrene, fluorene, chrysene and acenaphthylene while the least abundant were benzo(g,h,i) perylene, dibenz(a,h))anthracene, benzo(b) fluoranthene, and benzo(a) pyrene (Fig. 2; Supplementary Table 1). Overall, out of the 15 most abundant hydrocarbons analyzed, 11 of them had significantly higher composition in oil-polluted soil samples than in the control soil samples. Soil samples collected from industrial oil-polluted sites in Kampala had high contents for most of the different hydrocarbons.

### Bacterial community structure and activity

The study revealed significant variation in bacterial activity (Two-way ANOVA, F_(2,16)_ = 8.02, *p* = 0.004), bacterial species richness (Two-way ANOVA, *F*_(2,16)_ = 9.684, *p* = 0.0018) and diversity (Two-way ANOVA, F_(2,16)_ = 8.971, *p* = 0.002) amongst the sites. These bacterial species characteristics also varied significantly across the cities (Two-way ANOVA, F_(2,16)_ = 4.152, *p* = 0.035).

**Fig. 3:**
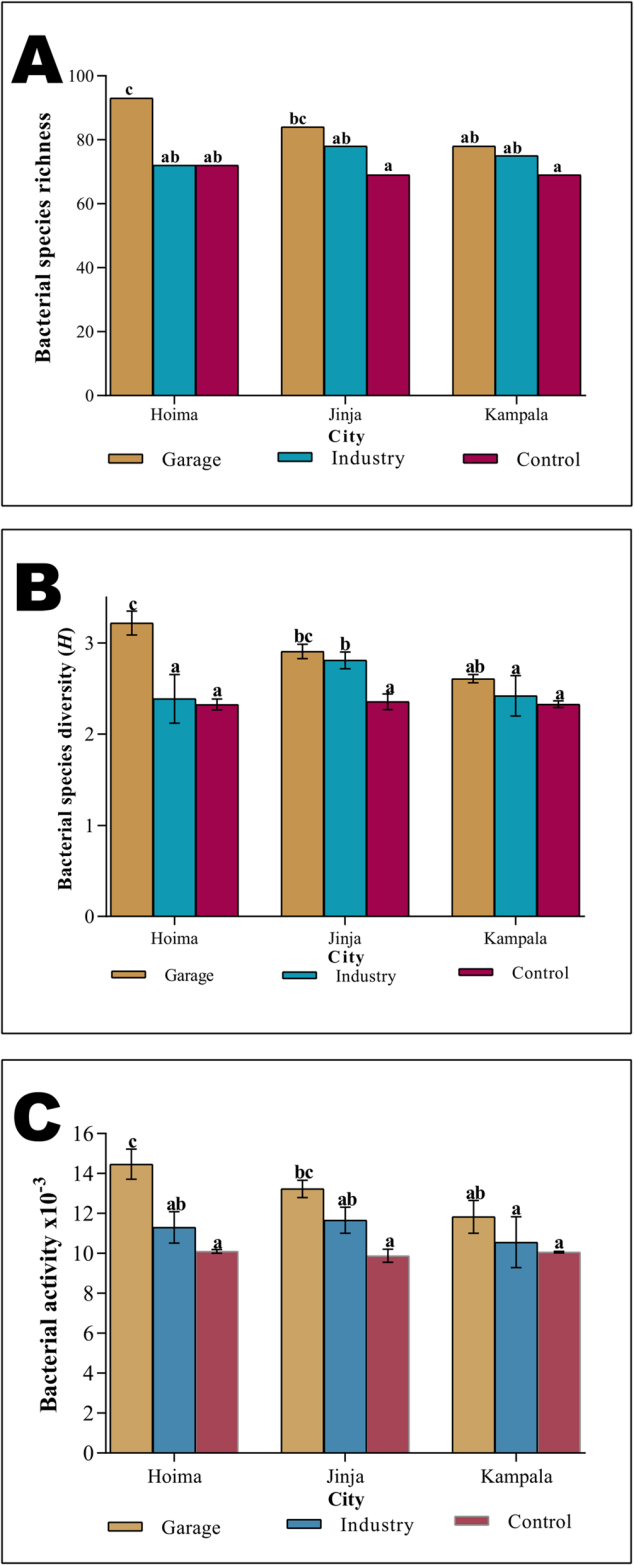
Bacterial species richness (A), bacterial species diversity (B) and bacterial activity (C) of the soil samples collected from the three cities. Means for each site for a particular city, with different letters of the alphabet are significantly different (Tukey’s test, *p* < 0.05)

Oil-polluted soil samples collected from garages had higher bacterial species richness, diversity and activity than that of the other sites for all the three cities. The two parameters, bacterial species richness and diversity did not show any significant variation across the three cities (ANOVA, *p* > 0.05). For Hoima city, the garage sites had significantly higher species richness, diversity and activity than the industrial and control sites, while for Jinja city, significant difference were only between garage sites and the control sites (Tukey’s test, *p* < 0.05). Contrary to what was observed in Hoima and Jinja cities, these bacterial population characteristics did not significantly vary for sites in Kampala (Tukey’s test, *p* > 0.05) (Fig. 3; Supplementary Table 1). Oil-polluted soils collected from garages in Hoima and Jinja were comparable (Tukey’s test, *p* > 0.05).

**Fig. 4:**
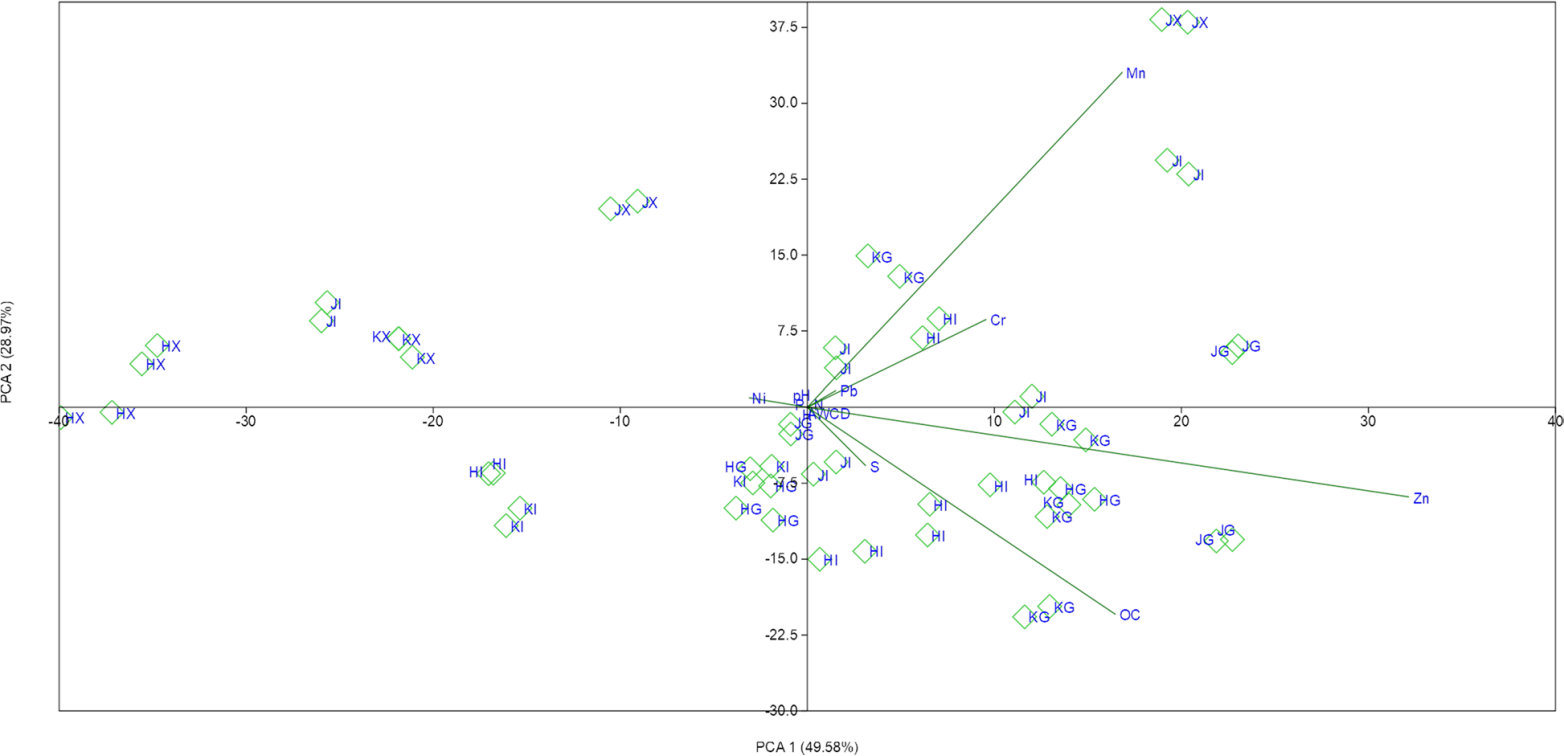
Principal component analysis biplots for oil-polluted and control soils collected from the three cities (KI, Kampala industry; KG, Kampala garage; KX, Kampala control; HI, Hoima industry; HG, Hoima garage; HX, Hoima control; JI, Jinja industry; JG, Jinja garage; JX, Jinja control)

The PCA biplot shows that PC 1 and PC 2 explains 78.55% of the variations in the bacterial community structure and activity of the oil-polluted and control soils, with the PC 1 and PC 2 explaining 49.58 and 28.97% of the variability, respectively. With the exceptional case of some garage oil polluted soils from Hoima and Jinja and industrial oil polluted soils from Kampala city, all the oil polluted samples were distributed along the positive terminal of the PC 1 axis along with Zn, Mn, Cr, Pb, and OM content (Fig. 4). The control soils were distributed along the negative terminal of PC 1 with Ni except for some control soils that were collected from Jinja. Along the PC 2, the largest proportion of the oil polluted soil samples were distributed on the negative terminal while the control soil samples on the positive terminal along with Cr and Mn.

The correlation analysis revealed significant moderate positive correlations between the bacterial community profiles and organic matter content and zinc (Table 2). Similarly, the correlation between nitrogen and AWCD was also moderate and significant. All the correlations between bacterial community profiles and P, Cr and Pb were insignificant and weakly positive. Similar correlations were observed between pH and *H*; pH and S; Mn and *H*; Mn and AWCD and Ni and *H*. On the other hand, the correlations between S and Mn; S and Ni; AWCD and pH and AWCD and Ni were insignificant and very weakly negative.

**Table 2:** Correlation between heavy metal content and bacterial profiles (Spearman’s, *n* = 27)

Profiles	pH	OC	N	P	Zn	Cr	Mn	Ni	Pb
*H*	0.07^NS^	0.52**	0.33^NS^	0.27^NS^	0.59**	0.30^NS^	0.25^NS^	0.07^NS^	0.36^NS^
S	0.13^NS^	0.51**	0.25^NS^	0.19^NS^	0.56**	0.29^NS^	-0.01^NS^	-0.19^NS^	0.18^NS^
AWCD	-0.02^NS^	0.61**	0.47*	0.28^NS^	0.71***	0.29^NS^	0.13^NS^	-0.15^NS^	0.33^NS^

Species diversity (*H*), Species richness (S), Bacterial activity (AWCD), *p* ≤ 0.05*, *p* ≤ 0.01**, *p* ≤ 0.001***, NS – Not significant

The correlations between the bacterial community profiles and the hydrocarbons were largely positively correlated apart from the correlations with Benzo(k) fluoranthene (Bl), Benzo(a) pyrene (Bp) and Benzo(g,h,i) perylene (Be) (Table 3). Positive correlations between bacterial community profiles and Naphthalene, Acenaphthene, Fluorathnthene and Indeno(1,2,3-cd) pyrene were all significant. For Acenaphthylene and Phenanthrene, they were only significant for AWCD while for Fluorene and Pyrene, they were significant for both S and AWCD and Dibenz(a,h) anthracene for only *H*. On the other hand, the negative correlations were only not significant for S and Benzo(g,h,i)perylene

**Table 3:** Correlation between the abundant hydrocarbons and bacterial profiles (Spearman’s, *n* = 27)

Profiles	Na	Ac	Ae	Fl	Ph	An	Fu	Py	Ch	Bf	Bl	Bp	Da	Ip	Be
***H***	0.46*	0.38^NS^	0.66**	0.36^NS^	0.39^NS^	0.26^NS^	0.47*	0.37^NS^	0.30^NS^	0.45^NS^	−0.63***	−0.59**	0.49*	0.60**	−0.49*
**S**	0.45*	0.40^NS^	0.70**	0.39*	0.39^NS^	0.23^NS^	0.43*	0.52*	0.45^NS^	0.60^NS^	−0.50**	−0.48*	0.46^NS^	0.48*	−0.34^NS^
**AWCD**	0.50**	0.49*	0.64*	0.49*	0.41*	0.30^NS^	0.51**	0.53*	0.39^NS^	0.62^NS^	−0.47*	− 0.43*	0.44^NS^	0.65**	−0.50**

Species diversity (H), Species richness (S), Bacterial activity (AWCD), *p* ≤ 0.05*, *p* ≤ 0.01**, *p* ≤ 0.001***, NS – Not significant, Naphthalene (Na), Acenaphthylene (Ac), Acenaphthene (Ae), Fluorene (Fl), Phenanthrene (Ph), Anthracene (An), Fluorathnthene (Fu), Pyrene (Py), Chrysene (Ch), Benzo(b) fluoranthene (Bf), Benzo(k) fluoranthene (Bl), Benzo(a) pyrene (Bp), Dibenz(a,h) anthracene (Da), Indeno(1,2,3-cd) pyrene (Ip), Benzo(g,h,i) perylene (Be)

## Discussion

We report the abundant presence of metabolically active indigenous bacterial species and the chemical characteristics of soils that have a long history of oil pollution in Uganda. Elsewhere, the bacterial species isolated from such oil-polluted soils have been proposed to potentially possess oil-degrading traits, and may therefore be used in the development of bacterial-based products for remediation of oil-polluted sites [22]. The findings of the current study are novel since, to the best of our knowledge, there is no earlier baseline research that confirmed the existence of potential petroleum hydrocarbon-degrading microbes in polluted soils in Uganda.

Our findings revealed that all the soil samples were characterized with median pH values that were neutral except for soil samples collected from industrial oil-polluted sites in Kampala. The median pH and pH ranges determined in this study for both oil polluted and control soils were within the ranges characterized to be neutral according to Jensen and Thomas [51], except for industrial oil polluted soil samples collected from Kampala city which had a weakly alkaline pH value of 7.9. In line with previous studies by Achuba and Peretiemo-Clarke [52], the changes in pH were minimal and within ranges that have no significant effects on the bacterial growth.

In agreement with earlier studies by Liao,Wang [53] and John,Ntino [54], we observed higher organic matter, nitrogen and phosphorus contents in the oil-polluted sites as compared to the control sites. This could be attributed to the presence of high organic matter, trace phosphorus and nitrogen in petroleum fuels [55–57]. Thus, the continuous pollution of the garage and industrial sites with petroleum fuels could have led to significant increase in the content of both organic matter and these nutrients, as reported elsewhere [58]. These results are partly in disagreement with the findings of Egobueze,Ayotamuno [59], who observed that petroleum fuel contamination increased the amount of organic matter but decreased the available phosphorous and nitrogen [59]. Though some researchers have reported that the high nutrient content of petroleum fuels supports the growth of oil degrading bacteria [60, 61], there is evidence that the alterations in soil properties resulting from oil pollution impair plant growth and other ecosystem services [62]. Therefore, the remediation of oil polluted soil to restore its natural state is vital.

We report a generally high concentration of heavy metals in the polluted sites than the control sites. Similar results were earlier reported elsewhere [63]. However, we observed that with the exception of zinc which had significant correlation with the bacterial community profiles (Table 2), the differences in the concentrations of the other heavy metals between the oil polluted and the control sites were not high enough to cause significant differences in bacterial community parameters. The co-existence of both oil-hydrocarbons and zinc, as observed in the current study, may pose threats to ecosystem health. In line with previous studies by Ijah and Antai [64], our study showed that there was excessively high concentrations of oil hydrocarbons in the oil-polluted sites as compared to the control sites. This could be explained by the fact that oil is rich in different types of hydrocarbons [65], and it therefore augments the total hydrocarbons present in the environments it pollutes.

Evident from the ecological analysis and PCA results, the bacterial community profiles between the oil-polluted and control sites were largely similar, suggestive of the occurrence of metabolically active bacterial populations in both sites. However, we observed significantly higher bacterial diversity, species richness and activity in oil-polluted garage soils than all the other sites. For the industrial oil-polluted sites, these bacterial community parameters were higher than that of the control, though not significantly different. Generally, the higher bacterial species richness and diversity in oil-polluted soil has also been reported earlier by Liao,Wang [53] and Ikhajiagbe and Ogwu [66]. The higher species richness and diversity reported in the current study are suggestive of the existence of different indigenous bacterial species with varying metabolic functions in oil-hydrocarbon biodegradation processes.

Despite this observation, the actual bacterial species present could be different between the oil-polluted and control soils, with the oil polluted soils harboring species with higher potential for oil degradation. The long exposure to significantly higher concentrations of oil hydrocarbons as observed in this study could have mediated the bacterial community shift towards more efficient oil degrading species. The shifts in microbial communities due to hydrocarbon exposure have also been pointed out in several studies [60, 67]. The shifts in bacterial composition due to oil pollution leads to the establishment of bacterial communities, of which potential oil-degraders constitute up to 100% [67]. However, the existence of oil degrading bacteria in unpolluted soils should not be overlooked as they have been reported to occur there-in [68–70]. The potential variations in the actual bacterial species between the oil-polluted and control soils in Uganda need to be ascertained.

## Conclusion

This study revealed largely similar bacterial community profiles between the oil-polluted and control sites suggestive of the occurrence of metabolically active bacterial populations in both sites. Additionally, we observed higher concentrations of hydrocarbons such as pyrene, anthracene and phenanthrene as well as heavy metals, organic matter content, nitrogen and phosphorus in the oil polluted sites than the controls. Even though we observed similar bacterial community profiles between the oil polluted and control sites, the actual bacterial community composition may be different, owing to a higher exposure to petroleum hydrocarbons. However, the existence of oil degrading bacteria in unpolluted soils should not be overlooked. Thus, there is a need to ascertain the actual indigenous bacterial populations with potential to degrade hydrocarbons from both oil-polluted and unpolluted sites in Uganda to inform the design and development of a bacterial-based oil remediation product that could be used to manage the imminent pollution from oil exploration and increased utilization of petroleum products in Uganda.

## Supplementary Information

**Additional file 1.**

## Data Availability

All data generated or analysed during this study are included in this published article [and its supplementary information files].

## Abbreviations

GC-MS: Gas Chromatograph-Mass Spectrophotometry
AWCD: Average Well Colour Development
OD: Optical density
UNCST: Uganda National Council for Science and Technology
DGAL: Directorate of Government Analytical Laboratory
